# Supplementary material for: Efficient Bayesian inference for mechanistic modelling with high-throughput data
Source: PLoS Comput Biol. 2022 Jun 21;18(6):e1010191. doi: 10.1371/journal.pcbi.1010191 (PMC9249175; doi:10.1371/journal.pcbi.1010191)
Supplement: Attachment — Submitted filename: Response_to_reviewers_EfficientBayesianInference.pdf [file pcbi.1010191.s002.pdf]

Mathematical Institute
Andrew Wiles Building, Radcliffe Observatory Quarter
Woodstock Road, Oxford, OX2 6GG

UNIVERSITY OF OXFORD

Professor Jason Papin
Editor-in-Chief
PLOS Computational Biology

5th April 2022

Dear Professor Papin,

Re-submission of manuscript PCOMPBIOL-D-22-00221

I am writing to submit the revised version of our manuscript "Efficient Bayesian inference for mechanistic modeling with high-throughput data" for consideration in *PLOS Computational Biology*.

We would to take this opportunity to thank the reviewers for their constructive comments. In our revised manuscript, we have carefully addressed all comments, with changes in the revised manuscript marked in purple.

We hope that you will now find our manuscript suitable for publication. Please do not hesitate to get in touch if you have any queries.

Best wishes,

Simon Martina-Perez
Doctoral candidate in mathematics
University of Oxford

General Enquiries Tel: +44 (0)1865 273525 Direct: +44 (0)1865 283893
Fax: +44 (0)1865 273583 Email: martinaperez@maths.ox.ac.uk Web: http://www.maths.ox.ac.uk/people/profiles/martinaperez

Efficient Bayesian inference for mechanistic modelling with high-throughput data

S. Martina-Perez, H. Sailem and R. E. Baker

Response to comments by Reviewer 1

The authors develop a new ABC method based on mini-batching (inspired by stochastic gradient descent in machine learning) in order to improve Bayesian inference for models with intractable likelihoods in the presence of a large number of independent datasets. Instead of trying to match on every dataset at each iteration, the comparison is based on a mini-batch of datasets randomly samples from all the datasets. The approach looks promising, but there are some issues/questions that need to be addressed before I can properly assess it:

We are glad that Reviewer 1 is supportive of publication of our manuscript. We would like to thank them for their constructive comments, which we respond to in full below.

As a general remark, we would like to clarify some of the terminology used by Reviewer 1, to ensure consistent use of terminology in this review and the manuscript. With 'dataset' we refer in the manuscript to all the data corresponding to one given gene knockdown, e.g. all the experimental data for CDC42 knockdown would be one dataset. With 'datapoint', we refer to one individual pair of initial and final condition for the same experiment. Therefore, we consider various different independent datasets (in this case 118), each with a varying number of datapoints. We remark that, rather unfortunately, the number of datapoints within the Mock dataset is 117, so we appreciate that there might be some confusion.

The reason we would like to make this clarification upfront is that Reviewer 1 remarks that "Instead of trying to match on every dataset at each iteration, the comparison is based on a mini-batch of datasets randomly sample[d] from all the datasets". However, this is not the case. It is the case that instead of trying to make a comparison based on every datapoint at each iteration, the comparison is based on stochastically sampled minibatches of datapoints (not datasets). Some of the datasets considered (for example Mock, CDC42, CDH5), contain many different datapoints, meaning that we choose a minibatch approach so that inference can be carried out efficiently for each one of these large datasets. Section 3.3 provides the precise details. We reiterate that, since we carry out ABC-SMC independently using a minibatch approach for each of the datasets, the result is 118 different posterior distributions, for 118 different datasets, each corresponding to a different gene knockdown.

1. Does the new approach outperform simply an analysis of a sensible subset of the dataset of the same size as the mini-batch? Eg some kind of stratified sampling to get a representative sample of initial conditions.

   We thank Reviewer 1 for this remark. We would like to stress that the point of using minibatches in ABC-SMC is to make use of the full data available in a computationally efficient way. The point of our approach is not to get a representative sample of initial conditions (or of anything else) by down-sampling. The problem with initial conditions in our work is that the variability in initial conditions between experimental replicates means that each model simulation must be initialised differently, leading to a huge computational burden. What the reviewer proposes is to a priori *take a subsample from the data and perform ABC by only using that part of the data. In order for this method to offer the same computational speed-up as minibatching, this amounts to taking a subset of the data that contains N_{bs} samples (e.g. to throw away 107 out of 117 observations in the Mock dataset). Throwing away a large part of the dataset would indeed reduce the number of simulations performed and hence the computational cost. However, this approach comes with several grave problems. For example, if the observed behaviours in a screen are very variable, getting rid of observations will be detrimental to the quality of the posterior as the ABC method will not learn from the full distribution of the data. In contrast, our minibatch approach uses all the data available, meaning that no bias is introduced into the results (see also our response to Reviewer 1's Question 4). At this point, we would like to highlight that the uncertainty quantification provided in Supplementary Information Section 4 deals with this problem by showing that the posterior distributions obtained using minibatch ABC-SMC recover the same behaviours as the posteriors obtained using traditional ABC-SMC, at a fraction of the computational cost.*

To answer the Reviewer's question systematically, we have tested the performance of subsampling. Below we copy the newly added test, which is now included in Supplementary Information Section 4.5.

To compare the performance of minibatch ABC-SMC with naive subsampling from the data, we fix $N_{\text{bs}} = 10$ as in the main text and investigate the quality of the posterior distributions obtained by performing ABC-SMC on subsamples of size N_{bs} of the data. From the Mock dataset, we select 100 datapoints at random and without replacement, and divide them into 10 subsets of $N_{\text{bs}} = 10$ datapoints. Like in Supplementary Information Section 4.4, we perform minibatch-ABC-SMC on each of the resulting *miniature data sets* and record their posterior distributions. We independently run 10 instances of of minibatch ABC-SMC, again using $N_{\text{bs}} = 10$, and record the posterior distributions. Figure S9 shows boxplots of the means of the posterior distributions found by performing ABC-SMC on subsets of the data as well as using minibatch ABC-SMC. The boxplots show that the subsample approach yields more variable posterior means than minibatching.

Figure S10 shows boxplots of the variance of the posterior distributions generated by performing ABC-SMC on subsets of the data as well as using minibatch-ABC-SMC. The boxplots show that the subsample approach yields on average larger posterior variance than than minibatching, indicating that the posterior obtained using subsampling contains more uncertainty than that obtained using minibatch ABC-SMC.

This has been summarised in the main text as follows:

At this point, we remark that an alternative to using minibatch ABC-SMC might be to *a priori* select a subset of the data (*e.g.*, using stratified sampling) and use ABC-SMC on this subset. While reducing the amount of data to be processed would certainly reduce the computational burden of ABC-SMC, this approach also vastly reduces the amount of available information. Moreover, subsampling *a priori* from the data might introduce bias into the problem, as it is up to the practitioner to define a sensible subset of the data. In Supplementary Information S4.5, we carefully examine the quality of the posteriors generated by subsampling from the data in comparison to those generated using minibatch ABC-SMC. We find that *a priori* selected subsets give rise to more variability in the estimated posterior mean, and higher posterior variance across all parameters, than minibatch ABC-SMC with minibatches of the same size. We conclude that our approach is to be preferred over simply reducing the amount of available information by subsampling the data.

To conclude, we agree with Reviewer 1 that it is a possibility to choose a subsample from the data and perform ABC-SMC on this subsample. However, this comes at the cost of more variable and less reliable results for the posterior than minibatching. Finally, we would like to stress that the decision to subsample from the data introduces bias into the problem and throws up a number of further questions. For instance, how does one even define what a 'sensible' subset of the data is? To attempt to answer this question, one must at the very least know how large the subsample must be, which entails choosing different subset sizes and analysing the performance of the method for each. In addition, one must analyse what the effect is on the variability of the posterior distributions when different subsetting rules are used. We remark that this approach would suffer from exactly the same problem as that identified by Reviewer 1 in their Question 2 (see below), meaning that it does not offer a straightforward alternative to our approach.

2. If the answer to 1 is yes, then the paper really needs to develop a principled method for selecting the mini-batch size for a given application. At the moment the paper tries a bunch of mini-batch sizes to see which is the best, but in practice if we try a bunch of different mini-batch sizes then we might lose any computational savings.

   We would like to thank Reviewer 1 for this remark. At this stage, we have, in fact, proposed a simple, yet principled method to choose the batch size (see Supplementary Information Section S4). This approach, to summarise the section, amounts to analysing the systematic error and variability of the estimated parameter when different batch sizes are used, and choosing a value of the batch size where the variability plateaus while not incurring systematic errors in estimating model parameters. We remark that this amounts to a grid search through the batch size hyperparameter. Performing a grid search through algorithm hyperparameters prior to using the method on experimental data is a very common method in computational science to choose hyperparameters, and

we believe that at this stage it is a sensible method to obtain rigorous uncertainty quantification in predictions (as understanding the performance of the method on synthetic data can help understand the ability of the method to perform on data). In future work, a different, perhaps more computationally efficient method, might be proposed, and we would welcome this approach.

The shortcoming of performing the hyperparameter grid search, as the reviewer notes, is that the computational cost of the grid search using different batch sizes might undo the computational gains from doing minibatch ABC-SMC. At present, we believe that this is a limitation of our method that can be addressed in future work. Nevertheless, we would like to point out that in applications like ours there may exist a need to perform ABC-SBC a great number of times. In our application, there are 118 datasets, each of which requires one ABC-SMC run. If in each such ABC-SMC run, the computational time is substantially reduced through the implementation of a minibatch approach, the computational savings can still greatly exceed the extra cost of the hyperparameter grid search (since one only needs to conduct the grid search for one, not all of the 188 datasets).

We do believe that this raises an important point regarding the applicability of our method, and we have added the following paragraph to our discussion section:

In this work we have used a simple method to choose the batch size, which amounts to analysing the systematic error and variability of the estimated parameter posterior distributions when different batch sizes are used, and choosing a value of the batch size where the variability plateaus while not incurring systematic errors in estimating model parameters. We remark that this amounts to a grid search through the batch size hyperparameter. Performing a grid search through algorithm hyperparameters prior to using the method on experimental data is a common approach in computational science to choose hyperparameters. In this case, the method yields additionally a rigorous uncertainty quantification of model predictions. The potential shortcoming of performing the hyperparameter grid search is that the computational cost of the grid search using different batch sizes might undo the computational gains from performing minibatch ABC-SMC. However, in many applications there may exist a need to perform ABC-SBC a great number of times. For example, in this work, there are 118 datasets, each of requires one ABC-SMC run. If, in each such ABC-SMC run, the computational time is substantially reduced (and we note that the speed-up reported for *e.g.* the Mock dataset was a factor 11), the computational savings can still greatly exceed the extra cost of the hyperparameter grid search. At present, our method offers a computational improvement in such a setting. In future work, a different, perhaps more computationally efficient, method for choosing the minibatch size might be proposed, which would greatly extend and improve the applicability of our work.

3. In the application the paper implicitly assumes that the same parameter value is responsible for generating all the datasets, but could there be any variation between parameter values for the different experiments? A related question is how well the model fits the data (including the datasets that were not included in the final mini-batch)? I don't think this was properly assessed in the paper.

   We thank Reviewer 1 for this remark. Firstly, as our responses to Question 1 hopefully have already clarified, we would like to highlight that all datapoints from a dataset are used to estimate the posterior distribution using the minibatch ABC-SMC approach, and so there is no "final minibatch".

   Reviewer 1 is indeed correct that we assume that the same parameter is responsible for generating all the observed data within a given gene knockdown dataset (and, correspondingly, that different gene knockdowns can be described using different parameters). More precisely, we assume that all the datapoints within any one dataset can be described using model outputs with the same parameter, but the different datasets (which each contain data for a different gene knockdown, see general remark) are of course described by model outputs with different parameters. On the assumption that within each gene knockdown we can describe the process using the same parameter, we note that the experiments were performed in 96-well plates to reduce the variability in experimental conditions, meaning that most data for a single gene knockdown share comparable experimental conditions that might influence the way in which gene-knockdown specific effects could be identified from the data. The reason that we use Bayesian inference in this work is furthermore to reflect the uncertainty in the information gained by the data.

   The reviewer's question about understanding how well the model fits the data is interesting, and we have added the following paragraph to the manuscript to show that model predictions generated

using the ABC posteriors are in excellent agreement with the experimental data.

Finally, we assess how well our IBM predicts experimentally observed wound healing behaviour when parametrised according to the posteriors generated using minibatch ABC-SMC. We sample 1000 parameters from the posterior distribution obtained using minibatch ABC-SMC with $N_{\text{bs}} = 10$ on the full Mock dataset and select one Mock initial condition uniformly at random. For this condition, we perform forward model simulations, one with each sampled parameter, and record the summary statistics defined in Section 2.3.3, which are the density profile, pair correlation function, and number of alive cells at $t = 24h$. From these forward simulations, we compute the mean and standard deviation for the density profile and pair correlation function. In Figure 3, we display the experimental data for density profile and pair correlation together with a range of one standard interval from the posterior mean. We also show a histogram of simulated cell counts at $t = 24h$, compared to the experimentally observed cell count at $t = 24h$. The posterior predictive ranges for the density profile and pair correlation are excellent fits to the data, and the simulated cell count histogram is centered around the experimentally observed cell count. *See added Figure 3.*

4. This mini-batching is a bit different to whats (*sic*) in stochastic gradient descent, since there all the proposed updates are accepted. Whereas here mini-batches are rejected if they dont (*sic*) generate a small discrepancy. So is there some bias in the datasets that end up being selected in the mini-batch, ie some are accepted much more than others, since they are "easier" to match on, but may not be as informative about the parameters?

   *We thank Reviewer 1 for this question. We would like to highlight that it is not the case that “mini-batches are rejected if they dont (*sic*) generate a small discrepancy.” Our minibatch-ABC approach uses a minibatch of the data (instead of the full data) to compute the distance between model simulations and data for each sampled parameter, whereas in stochastic gradient descent a minibatch (instead of the full data) would be used to estimate the gradient. The ‘update’ in stochastic gradient descent is to use this gradient to change the value of some quantity of interest (for example, network parameters in a neural network), whereas, to use the reviewer’s analogy with stochastic gradient descent, the ‘update’ done with ABC (both minibatch and standard) is to make a decision whether to keep a given parameter for the next generation, or not. It is the error estimate based on the minibatch that is used to form the new generation. Since each minibatch in the data is treated equally to make this ‘update’ to the previous generation, there is no bias in the data points that are used to assess the fit between model and data. Our computation in response to Reviewer 2’s Question 2 shows that every datapoint will be included (with exceedingly high probability) at some point during the inference process. The conclusion of this is that the parameters that are accepted into the posterior distribution need to fit the data well, and that if a parameter yields model simulations that are far away from one of the datapoints in a minibatch, it will not be accepted into the posterior distribution.*

Response to comments by Reviewer 2

The authors proposed an adaptation of the concept of mini-batching, which is widely used in the area of machine learning and which starts findings its way into other applications, to Approximate Bayesian Computation, with particular emphasis to systems biology. The method is evaluated on a stochastic model of single-cell motility and proliferation, which is used to model and understand a high-throughput scotch assay experiment.

I consider this a valuable contribution, as the availability of measurement data using high-throughput methods is indeed blessing and curse at a time. Learning from huge amounts of data is complicated and necessitates the development of new methods. ABC seems to be a methods which is particularly suited for combining it with mini-batching, due the stochastic nature of both concepts. The application example is interesting and important and showcases the potential of the method, although it is probably an edge case: It is just about to be large enough to see benefits from using a mini-batch approach, but not yet large enough to make mini-batch sampling the only way to cope with it (and the authors also don’t claim that).

I feel like the manuscript is a bit in between a pure method contribution and an actual biological application. This has the advantage of showing a biologically relevant application example, but comes with the downside that e.g. the methodological part is not as thoroughly worked out as it could be the case.

I have a few (mostly technical) points, of which I hope they will help to improve the manuscript.

1. I think the speed-up in lines L.376 - L.378, L. 411 is computed incorrectly. The authors run 4 generations of ABC-SMC, with mini batch size 10. That means only 40 datapoint[s] (i.e., initial conditions) out of 118 are used at all. Typically, one uses in the mini batch methodology the notion of „epochs“, where one epoch is one pass through the dataset. In the present example, the model was not even „trained“ on the full dataset, so it was trained for less than one epoch. It is thus misleading to compare the computational speed-up with a full-batch approach using the whole dataset, since the model has not been informed by the full dataset. An approach using less than one epoch may work for this particular model and this particular dataset, but I doubt that this is a reliable approach in general. I would rather expect that for a general mini-batch-ABC-SMC application using N generations, the computational speed-up cannot exceed N, or, in this case, 4. It would be good if the authors used the notion of epochs and elaborated on this effect a bit.

   We thank Reviewer 2 for this query. We believe, however, that our computations are correct. The reviewer bases their analysis on the claim "The authors run 4 generations of ABC-SMC, with mini batch size 10. That means only 40 datapoint[s] (i.e., initial conditions) out of 118 are used at all." However, this assertion is incorrect. Using the notation from the manuscript, within each of the N ABC-SMC generations, there are N_{gen} parameters that are sampled, of which N_{acc} are accepted into the next generation. Within standard ABC-SMC, for each of the N_{gen} parameters, one simulation for each of the N_{obs} datapoints needs to be made (see Equation (8) of the manuscript), so a total of $N_{gen} \cdot N_{obs}$ simulations are performed within each generation. With minibatch ABC-SMC, for each sampled parameter individual simulations are only performed for the N_{bs} stochastically sampled datapoints, rather than all $N_{obs} \gg N_{bs}$ datapoints. This means that (again referring to Equation (9)) a total of $N_{gen} \cdot N_{bs}$ simulations are performed. Hence, in total, ABC-SMC without minibatching performs N_{obs}/N_{bs} more simulations than ABC-SMC with minibatching. Assuming (as in the manuscript) that on average all simulations are equally expensive, that means that on average, the computational time for minibatch ABC-SMC in each generation is a fraction N_{obs}/N_{bs} smaller, meaning that the total computational time (which is N times the computational time per generation) is also a fraction N_{obs}/N_{bs} smaller. This is indeed what the computations in our methods and results sections reflect.

   To address the reviewer's concerns about the concept of epochs, we would like to point out that this terminology comes from machine learning (not "mini-batching" more generally) and does not carry any meaning within the context of ABC, so the term cannot be directly applied in this context. ABC-SMC does not "train" the model by doing a "pass through the dataset" at each generation. Rather, in ABC-SMC there is a population of parameters at various generations, which are individually evaluated by comparing model outputs generated using that parameter with all of the available data. In other words, if there are N_{gen} sampled parameters in each generation, there will be N_{gen} "passes through the data" in each of the N generations. If an epoch is defined as a "pass through the data", then within standard ABC-SMC there will be N_{gen} such passes in each generation (and it might be helpful to recall that in this application, $N_{gen} = 2 \cdot 10^4$). In conclusion, one generation of standard ABC-SMC does not correspond with the concept of epoch as defined by the reviewer.

   The statement by the reviewer "an approach using less than one epoch may work for this particular model and this particular dataset, but I doubt that this is a reliable approach in general" reflects a valid fear that not all datapoints will be used in one minibatch ABC-SMC generation. With our minibatch approach, there is a pass through a subset of size N_{bs} of the data for each of the N_{gen} parameters. Since for each of the N_{gen} parameters N_{bs} datapoints are sampled uniformly at random with replacement, we can compute the probability that one generation of minibatch ABC-SMC fails to contain a given datapoint, say $i_\star \in \{1, \ldots, N_{obs}\}$. This is equivalent to finding the probability that $i_\star$ is not chosen within $N_{gen} \cdot N_{bs}$ draws with replacement from the set $\{1, \ldots, N_{obs}\}$. Calling this event $E_{i_\star}$, a simple computation shows that this probability is given by

$$\mathbb{P}(E_{i_\star}) = \left(1 - \frac{1}{N_{obs}}\right)^{N_{gen} \cdot N_{bs}} \leq e^{-(N_{gen} \cdot N_{bs})/N_{obs}}, \tag{1}$$

   since $(1 + x/n)^n < e^x$ for any integer n. Given that in our work $N_{gen} = 2 \cdot 10^4$, $N_{bs} = 10$ and

$N_{obs} = 117$, *we conclude that*

$$\mathbb{P}(E_{i_\star}) \leq \textit{exp}\left(-\frac{2 \cdot 10}{117} \cdot 10^4\right) = \textit{exp}(-1.6 \cdot 10^3), \tag{2}$$

meaning that the event that in one generation not all datapoints are used is essentially negligible. To use the reviewer's terminology, each generation will correspond to at least one epoch, in the sense that in each generation, with very high probability, all datapoints will be used at least once by the minibatch ABC-SMC algorithm. We believe that this is an important concern to highlight, and we have added the following text to the end of Section 2.3.2 so that readers are aware of this.

We note that the random sampling of minibatches ensures that with very high probability every datapoint in the dataset will be used in each generation. Since for each of the N_{gen} parameters in each generation there are N_{bs} datapoints sampled uniformly at random, the probability that one generation of minibatch ABC-SMC fails to contain a given datapoint, say $i_\star \in \{1, \ldots, N_{\text{obs}}\}$, can be computed. Denoting this event $E_{i_\star}$, a simple computation shows that this probability can be bounded by

$$\mathbb{P}(E_{i_\star}) = \left(1 - \frac{1}{N_{\text{obs}}}\right)^{N_{\text{gen}} \cdot N_{\text{bs}}} \leq e^{-(N_{\text{gen}} \cdot N_{\text{bs}})/N_{\text{obs}}}, \tag{3}$$

since $(1 + x/n)^n < e^x$ for any integer n. For $N_{\text{gen}} = 2 \cdot 10^4$,

$$\mathbb{P}(E_{i_\star}) \leq \text{exp}\left(-\frac{2 \cdot N_{\text{bs}}}{N_{\text{obs}}} \cdot 10^4\right), \tag{4}$$

meaning that the event that in one generation not all datapoints are used is essentially negligible.

2. The authors use ABC-SMC with a constant acceptance rate (fixed to 2,5%), which is not a common approach, I think. Usually, acceptance rates or effective sample sizes are adapted (e.g., decreased over time) in order to converge to the true posterior distribution. Different approaches how to choose the samples for the next generations exist (see e.g. [1], [2] or [3]). I would be interested if the approach used by the authors can converge to the true posterior at all (e.g., if there is a mathematical proof for this, ideally including the mini batch adaptation of the method). Alternatively, it might be worthwhile to test the minibatching approach on a (smaller) toy model and compare it to a well established method or (if possible) a ground-truth. In am particularly interested in this, as the number of generations used in this example seems comparably low to me.

   Firstly, as for the number of generations, we would like to highlight that we performed minibatch ABC-SMC with a larger number of generations, and found that including more generations than $N = 4$ did not lead to a significant change in the posterior. This analysis can be found in the Supplementary Information.

   Further, we would like to point out that ABC-SMC with a constant acceptance rate is, in fact, a common approach to choosing the sampling schedule in ABC, and these methods are often referred to as quantile-based. Marin et al. provide a discussion in [4]. The quantile-based approach has been widely recognised as a straight-forward and intuitive approach to choosing the parameters for the next ABC generation, see for example [5, 6, 7], which all use this scheme explicitly. In the literature, some ABC-SMC approaches are described as depending on a decreasing sequence of tolerance levels $\{\epsilon_i\}$. However, the choice of such tolerance levels is difficult in practice, and has been met with the challenge of finding a principled way of finding the optimal tolerance level at each generation [8]. In this context, the justification for choosing a set of tolerance levels has been to choose a set of tolerance levels such that from each generation to the next, a fixed proportion of parameters is accepted into the following generation [4, 8]. So, it is common for ABC-SMC implementations to use a constant acceptance rate as an implementation tool, either explicitly, or implicitly, and in fact choosing a sequence of tolerance levels that is decreasing and choosing a sequence of acceptance rates to accept at each new generation is equivalent.

   Strikingly, all of the works cited by Reviewer 2 propose a scheme to adaptively set the tolerance levels by methods geared towards achieving target acceptance rates. We commence with Silk et al. [2], who develop a method explicitly based on achieving an acceptance rate with some predetermined properties. This method seeks to generalise quantile-based approaches, and the conclusion of the paper is that this method "performs comparably to the best fixed quantile schedules" (Caption of Figure 5) without out-performing them. With other words, this approach converges to

the posterior in their problems like the adaptive method does. The work cited by Schälte et al. [3] explicitly states that "the idea of this scheme is to match a specified target acceptance rate" (Section 2.7.1) as one of the principled approaches to choose how to design the next generation of parameters and they find that this method performs well in terms of the ability to recover the posterior and computational gains. Finally, the article by Del Moral et al. [1] uses the "fraction of alive particles" at the last generation (defined above Equation (13) in [1], which depends on ϵ_n) to find the tolerance level at the next generation, ϵ_n such that it satisfies the following criterion:

$$\mathit{PA}(\{W_n^{(i)}\}, \epsilon_n) = \alpha \mathit{PA}(\{W_{n-1}^{(i)}, \epsilon_{n-1}), \tag{5}$$

where W_n is the importance sample at generation n, and $\mathit{PA}(\{W_n^{(i)}\}, \epsilon_n)$ is the "fraction of alive particles" [1]. Note crucially that $\alpha \in (0, 1)$ is a pre-set (not adaptive) algorithm parameter. In words, this work produces adaptively a sequence of decreasing tolerance levels $\{\epsilon_i\}_{i=1}^T$ such that the number of alive particles at the next generation is a constant fraction of the previous generation, giving again a quantile-based approach.

We conclude that ABC-SMC implementations that use fixed acceptance rates from one generation to the next (either explicitly or implicitly) are well-established and standard and that many 'adaptive' schemes in the literature (including two out of three cited by the reviewer) explicitly use constant acceptance rates as the criterion for choosing the parameters to accept into the new generation.

3. At least I am not aware of minibatch approaches for ABC-SMC. However, minibatching has already been used for Bayesian methods such as MCMC (see [9]). Therefore, I think the work by Seita et al. is worth being mentioned by the authors, as it is on one hand close to their work, and on the other hand of interest for readers of this manuscript.

   We thank Reviewer 2 for making us aware of this interesting work. We have added a reference to this work in the introduction, after mentioning the application of minibatching in the context of ordinary differential equation models. The added sentence in the manuscript reads: and by Seita *et al.* [9] within the context of MCMC, likewise with a significant computational speed-up.

4. How are the mini batches drawn across different generations? Might it be possible to have one out of the 118 initial conditions used twice, despite using a mini batch size of 10 only?

   We thank Reviewer 2 for this question. The definition of how the samples are drawn is given in line 5 of Algorithm 1 in the manuscript. Each index for a minibatch sample, ξ, is drawn uniformly at random with replacement from the set $\{1, \ldots, N_{obs}\}$, where we recall that N_{obs} is the total number of observations. This means it is indeed possible to have one of the 118 initial conditions used twice. Assuming that some index, $i_\star$, has been drawn for the first sample of the minibatch, the probability that $i_\star$ will then be drawn at least once more in the minibatch is equal to $1 - ((N_{obs} - 1)/N_{obs})^{N_{bs}}$ which, given $N_{bs} = 10$ and $N_{obs} = 117$, is approximately equal to 0.07. Hence, on average, 93% of batches will contain 10 unique samples. In fact, there is no issue with this re-use of data: the model is stochastic and individual model runs can be calibrated to the data without issue.

5. On what infrastructure were the computational studies carried out? Was it a laptop or (as according to the acknowledgements) a cluster? How many nodes/cores were used and which types?

   We thank Reviewer 2 for this question. We have added a paragraph in the main text to address our infrastructure. This paragraph reads: All simulations were carried out using a development branch of the parallel open source ABC-SMC software Pakman [10]. Each ABC-SMC experiment was performed on a high performance compute (HPC) cluster using five 48-core Cascade Lake (Intel Xeon Platinum 8268 CPU @ 2.90GHz) nodes.

6. If possible, I would like to know how the computational speed-up reported by the authors compares to using established ABC-SMC toolboxes, which often allow for sophisticated parallelisation schemes, such as [5] or [6]. N.B.: Even if the speed-up by the method proposed by the authors does not exceed the one which can be achieved by using another toolbox, this does not reduce their contribution, as I consider the method being worth published also in that case, since it's original and could additionally combined with other, already existing concepts for efficient computation.

   We thank the reviewer for this comment. We would like to reiterate that our implementation uses Pakman [10], a parallelised ABC-SMC implementation that is benchmarked for performance on multiple computing architectures. Parallelisation reduces the computational time of ABC massively,

which is the reason why all our computations are parallel: the implementation in this paper is thus already a combination of parallel ABC with minibatching, rather than minibatching as an alternative to parallelisation. Put differently, the computational speed-up of our method is due to the reduced number of total computations that need to be carried out, while parallel computing makes use of distributed processing to carry out any fixed number of computations more quickly. This means that our method is not a replacement, or alternative, for parallelisation, and instead offers an improvement. Any parallelisation scheme will benefit from minibatching, since there will be fewer computations to distribute.

7. L. 32 - L. 35: Is this really an exponential increase? It is not clear to me why this has to be the case... Would be happy if the author could add a reference for this. Alternatively, they could reword this part.

   We thank the reviewer for this question. Given that the volume (Lebesgue measure) of a unit ball in n-dimensional space, V_n, is given by

   $$V_n = \frac{\pi^{n/2}}{\Gamma(n/2+1)} \sim \frac{1}{\sqrt{n\pi}} \left(\frac{2\pi e}{n}\right)^{n/2}, \tag{6}$$

   under uniform sampling, the probability of sampling a given volume around a given parameter will decay exponentially as the dimension of the parameter space increases. It is true, however, that we are not sampling uniformly at random in parameter space, so this estimate might indeed be too crude. It is, however, well-known that the computational effort of likelihood inference scales very poorly with the dimension of the parameter space [11, 12]. Therefore, we have changed the wording in these lines to:

   This means that large amounts of computationally costly simulations are required to compare model outputs with experimental observations, which is complicated further when the parameter set of the model is high-dimensional, as the cost of accurate likelihood estimation scales poorly with the dimension of the parameter space [11, 12].

8. I think the font sizes in the figures labels and legends should be increased in general, as they are sometimes hard to read and there is usually enough space to do so.

   We thank the reviewer for this remark. We have increased the font size and made all font sizes uniform across the figures.

9. In L.498, the authors speak of high-dimensional data. This feels a bit bizarre if the number of dimensions is five... The method used by the authors, which I think is appropriate, is also used on data with multiple hundred or thousand dimensions, where I consider this wording more appropriate. I would suggest to reformulate the sentence.

   We appreciate the reviewer's comment. While it is true that there are higher-dimensional problems one can apply K-means clustering to, the point is that for anything above dimension four it is difficult to visually inspect the spatial structure of the data. We have reworded, thus, to:

   One popular method of understanding the spatial structure of data, when it cannot be readily visualised due to the dimension of the data space, is K-means clustering.

10. In L.510, the authors state that the „elbow method“ is commonly used in data science. In that case, I would like to see a reference.

    We thank Reviewer 2 for this comment. We have added a reference to Nainggolan et al. [13], where the use of the elbow method is discussed.

11. Caption of Figure 10: The star, diamond, and plus are mentioned, but not shown in the figure.

    We thank Reviewer 2 for spotting this. The figure has been updated.

12. The reference to Supplementary information S3 is incorrect, I think: This should be S2.3 instead.

    We thank Reviewer 2 for spotting this typo. It has been fixed accordingly.

13. Typo in the Abstract: „but this measurements“ -> „but these measurements“

    We thank Reviewer 2 for this comment. We have corrected the typo.

References

[1] Del Moral P, Doucet A, Jasra A. An adaptive sequential Monte Carlo method for approximate Bayesian computation. Statistics and Computing 1–12 (2011).

[2] Silk D, Filippi S, Stumpf MPH. Optimizing threshold-schedules for sequential approximate Bayesian computation: applications to molecular systems, 2013. Statistical Applications in Genetics and Molecular Biology

[3] Schälte Y, Hasenauer J. Efficient exact inference for dynamical systems with noisy measurements using sequential approximate Bayesian computation, 2020. Bioinformatics 36

[4] Marin JM, Pudlo P, Robert CP, Ryder RJ. Approximate Bayesian computational methods. Statistics and Computing. 2012 Nov;22(6):1167-80.

[5] Beaumont M A, Cornuet J M, Marin J M, Robert C P. Adaptive approximate Bayesian computation. Biometrika 96, 983–990 (2009).

[6] Lenormand M, Jabot F, Deffuant G. Adaptive approximate Bayesian computation for complex models. arXiv preprint arXiv:1111.1308 (2011).

[7] Drovandi C C, Pettitt A N. Estimation of Parameters for Macroparasite Population Evolution Using Approximate Bayesian Computation. Biometrics 67, 225–233 (2011).

[8] Forrow A, Baker RE. Measuring the accuracy of likelihood-free inference; 2021. Available from: https://arxiv.org/abs/2112.08096.

[9] Seita D, Pan X, Chen H, Canny J An Efficient Minibatch Acceptance Test for Metropolis-Hastings 2018 Proceedings of the Twenty-Seventh International Joint Conference on Artificial Intelligence

[10] Pak T, Baker R, Pitt-Francis J. Pakman: a modular, efficient and portable tool for approximate Bayesian inference The Journal of Open Source Software 2020 Mar;5(47):1716.

[11] Järvenpää M, Gutmann MU, Pleska A, Vehtari A, Marttinen P. Efficient acquisition rules for Model-Based Approximate Bayesian computation. Bayesian Analysis. 2019;14(2):595 – 622. doi:10.1214/18-BA1121.

[12] Barber S, Voss J, Webster M. The rate of convergence for approximate Bayesian computation. Electronic Journal of Statistics. 2015;9(1):80 – 105. doi:10.1214/15-EJS988.

[13] Nainggolan R, Perangin-angin R, Simarmata E, Tarigan AF. Improving the performance of the K-means cluster using the sum of squared error (SSE) optimized by using the Elbow method. Journal of Physics: Conference Series 2019 Nov 1 (Vol. 1361, No. 1, p. 012015). IOP Publishing.
